# Is Hearing Impairment Associated With Specific Behavioural and Psychological Symptoms of Dementia in Japanese Older Adults Attending a Memory Clinic?

**DOI:** 10.1111/psyg.70090

**Published:** 2025-09-02

**Authors:** Saki Tanaka, Ryo Katayose, Teruaki Kawasaki, Ichiro Akiguchi, Mihoko Ogita

**Affiliations:** ^1^ Department of Clinical Nursing Shiga University of Medical Science Shiga Japan; ^2^ Department of Nursing Kyoto College of Nursing Kyoto Japan; ^3^ Kyoto Clinical and Translational Research Centre for Neurocognitive Disorders Kyoto Japan

**Keywords:** anxiety, dementia, hearing, hoarding, memory clinic, quality of life

## Abstract

**Background:**

Behavioural and psychological symptoms of dementia (BPSD), encompassing disturbances in perception, thought, mood and behaviour, are prevalent among individuals with dementia and can substantially affect their quality of life. Although prior research has suggested that hearing impairment (HI) may exacerbate BPSD due to communication difficulties and diminished social engagement, the relationship between HI and specific BPSD manifestations remains insufficiently explored. This study aims to examine the association between HI and distinct BPSD in individuals with cognitive decline attending a memory clinic.

**Methods:**

This study included 179 individuals with cognitive decline and their accompanying persons who visited a memory clinic between July 2023 and March 2024. Pure‐tone audiometry and a questionnaire survey were conducted. HI was defined as an average hearing level of 40 dB or higher. BPSD was assessed using a questionnaire derived from the BPSD25Q, a 25‐item questionnaire designed to evaluate BPSD. The association between HI and BPSD was analyzed using modified Poisson regression, with adjusted risk ratios (aRR) and 95% confidence intervals (CI) calculated.

**Results:**

A total of 144 individuals were included in the analysis, with a mean age of 82.7 years, approximately 70% of whom were female. The aRR (95% CI) for the presence of specific BPSD among individuals with HI were as follows: anxiety, 2.08 (1.09–3.99); irritability, 1.73 (1.05–2.86); verbally aggressive behaviour, 3.41 (1.24–9.40); physically aggressive behaviour, 5.54 (1.31–23.40); leaving home unannounced, 4.22 (1.25–14.16); hoarding behaviour, 1.83 (1.06–3.16); resistance to care, 2.12 (1.02–4.40); and carelessness with fire, 4.46 (1.12–17.83). No significant association was observed between HI and other BPSD.

**Conclusions:**

These findings suggest that HI may contribute to the presence of specific BPSD. Tailored interventions incorporating communication strategies and environmental modifications may help mitigate BPSD in individuals with cognitive decline and HI.

## 1 Introduction

Behavioural and psychological symptoms of dementia (BPSD) are characterised by disturbances in perception, thought content, mood, or behaviour that frequently occur in individuals with dementia [1]. These symptoms are highly prevalent, affecting 75% of patients aged 65 years and older with clinically diagnosed dementia who participated in the Cardiovascular Health Study in the United States [2] and ranging from 60% to 100% in long‐term care facilities across Asian countries [3]. Among the various BPSD subtypes, apathy, depression and agitation/aggression have been observed in more than 30% of cases [2]. The clinical significance of BPSD extends beyond the symptom burden, as specific symptoms, such as delusions, hallucinations, agitation and aggression, have been associated with increased mortality in individuals with dementia [4]. Given the substantial impact on patient outcomes, identifying and understanding the factors that contribute to the development of BPSD is crucial for effective clinical management.

Among the various factors associated with BPSD, social relationships and sensory function have emerged as particularly important contributors. Prior cross‐sectional studies have indicated that loneliness serves as a predictor of certain symptoms, including delusions, hallucinations and depression, in individuals with dementia [5]. Research in long‐term care facilities has further demonstrated that infrequent participation in activities, strained relationships with other residents and reduced communication with family members are associated with increased BPSD severity [6]. Building on this understanding of social factors, sensory impairments—particularly age‐related deterioration in vision and hearing—have been increasingly recognized as significant contributors to the development and severity of BPSD. Moreover, sensory function decline, including age‐related deterioration in vision and hearing, has been implicated in the occurrence and severity of BPSD. Notably, older adults with hearing impairment (HI) may be at heightened risk of developing and experiencing severe BPSD due to communication difficulties and diminished social engagement. Previous studies have demonstrated that among individuals with HI, those living alone or lacking social interactions were more likely to exhibit a higher number and severity of BPSD. Conversely, even individuals with HI who resided with family members or engaged in daily conversations with others displayed a greater number of BPSD. These findings suggest that it is not HI alone but rather the complex interplay of social and environmental factors within hearing‐impaired populations that influence the manifestation and severity of BPSD [7]. This suggests that HI has a direct effect on cognitive and behavioral symptoms in addition to compound social risk factors, making it a critical target for understanding the mechanisms of BPSD.

Previous studies examining the association between hearing loss and individual BPSD have reported that among individuals aged 49–93 years who visited a memory clinic, those with HI exhibited a higher prevalence of hallucinations, agitation/aggression, euphoria, night‐time behaviour disturbances and appetite changes compared to those without hearing loss, even after adjusting for demographic and clinical characteristics [8]. However, existing research has significant limitations that curb our understanding of this relationship. First, few studies have systematically investigated the relationship between HI and individual BPSD symptoms, with most focusing on composite BPSD scores [7, 8]. Although emerging research suggests a possible association between HI and BPSD as a whole, examining individual BPSD symptoms is crucial for clinical practice. BPSD encompasses a diverse range of symptoms, including agitation, depression, anxiety, irritability and resistance to care, each requiring distinct therapeutic approaches and management strategies. For instance, irritability may benefit from environmental modifications and communication strategies, while resistance to care often requires personalised behavioural interventions and caregiver training. Furthermore, understanding the specific symptom profile associated with HI can enable healthcare providers to implement targeted preventive measures and develop individualised care plans that address the most likely manifestations of BPSD in this population. This symptom‐specific approach has the potential to improve treatment outcomes, reduce caregiver burden and enhance quality of life for both patients and their families. Second and more importantly, no studies have conducted analyses that adequately adjust for factors unique to populations with HI, such as communication strategies, social support systems and environmental modifications that may modify the HI–BPSD relationship. This study aims to address these gaps by examining the association between HI and individual BPSD symptoms in individuals with cognitive impairment attending a memory clinic, whereas carefully adjusting for relevant demographic, clinical and HI‐specific factors.

## 2 Methods

### 2.1 Participants

**FIGURE 1:**
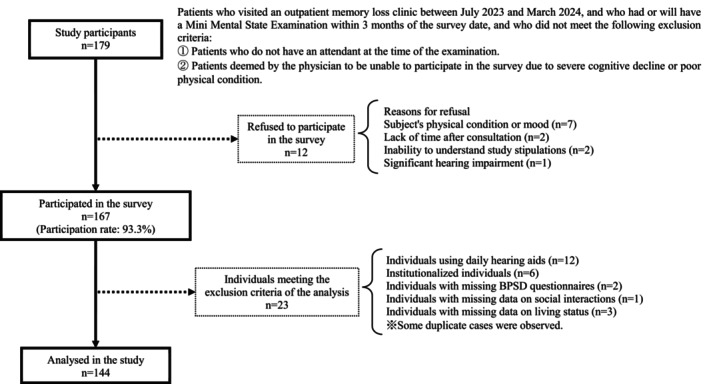
Flowchart of the study participants.

Between July 2023 and March 2024, this study recruited patients from an outpatient memory clinic, accompanied by a family member or caregiver who could provide collateral information and who had completed neuropsychological assessments within 3 months of the study period. Patients were excluded if they attended the clinic unaccompanied or had severe cognitive decline that precluded meaningful participation, as determined by the attending physician [7]. A total of 179 patient‐attendant pairs met the eligibility criteria. Of these, 167 provided informed consent (response rate: 93.3%), whereas 12 declined participation due to reasons such as poor physical or mental health, lack of time, difficulty understanding the research content, or significant HI (Figure 1). All participants underwent a pure‐tone hearing test and a questionnaire by interview. The researchers verified the consistency of questionnaire responses by consulting the attendants accompanying the patients, such as family members or friends. Furthermore, patient attendants completed a questionnaire on BPSD. A pure‐tone hearing test and a questionnaire interview were administered by pre‐trained licensed nurse researchers following the survey protocols.

### 2.2 HI

Hearing levels were evaluated using an audiometer (YAM1; Yagami Corporation, Aichi, Japan) to administer air‐conducted hearing tests. The speech‐frequency pure‐tone average (PTA) was determined by calculating the average minimum audible values at four conversational frequencies (0.5, 1, 2 and 4 kHz) and then summing these values and dividing them by 4. HI was defined using a cut‐off value of ≥ 40 dB in the better‐hearing ear, an established criterion for hearing loss known to significantly impact daily communication according to the Japan Audiological Society. Trained researchers conducted hearing tests in a quiet and private room with a background noise level of 35.1 dB, measured as an A‐weighted sound pressure level. To ensure accurate hearing assessment, participants were given the option to indicate their responses by either pressing a button or raising their hands, with the choice made to optimize test precision while maintaining response accuracy.

### 2.3 Assessment of BPSD

The BPSD questionnaire was developed based on the BPSD25Q [9], which comprehensively assessed the presence and severity of BPSD over the past month. The questionnaire included 25 symptoms, categorized into psychological and behavioral domains. Psychological symptoms included hyperactive symptoms such as visual and auditory hallucinations, delusions and day‐night reversal and hypoactive symptoms such as depression, anxiety and apathy. Behavioral symptoms were similarly classified into hyperactive symptoms, including stereotyped behavior, disinhibition, irritability, verbally and physically aggressive behavior, wandering, restlessness, leaving home unannounced, inappropriate sexual behavior, repetitive questioning, hoarding behavior, inappropriate vocalization and resistance to care and hypoactive symptoms such as lack of interest, withdrawal, excessive somnolence, poor hygiene, carelessness with fire and hiding or misplacing objects.

### 2.4 Other Variables

Data on age, sex, educational history, MMSE score, dementia type, living status, medical history and the number of psychotropic medications (including selective serotonin reuptake inhibitors [SSRIs], non‐SSRIs, typical and atypical antipsychotics and antiepileptic drugs) were collected from medical records. Dementia types were obtained from physicians' medical records and classified into mild cognitive impairment (MCI), Alzheimer's disease (AD) and other forms of dementia, such as vascular dementia, mixed dementia and dementia with Lewy bodies. Among the dementia types, AD was further categorised into Alzheimer's disease, suspected Alzheimer's disease, amnestic MCI and MCI due to Alzheimer's disease under the supervision of a dementia specialist. Social interaction, frequency of daily conversations and social isolation were assessed using self‐reported questionnaire responses. Social interaction was measured based on participation in community activities at least once a week, including neighbourhood association activities, local events, senior clubs and day‐care services. The frequency of daily conversations was assessed with the question: ‘How often do you talk to someone?’ Responses were categorised as follows: ‘1–5 days a week,’ ‘1–3 days a month,’ and ‘less than 1 day a month’ were grouped as ‘no conversation,’ whereas ‘almost every day’ was categorised as ‘daily conversatio.’ Social isolation was assessed using the Japanese version of the abbreviated Lubben Social Network Scale (LSNS‐6) [10], an internationally recognised tool for screening social isolation. The scale ranges from 0 to 30 points, with higher scores indicating a wider social network. The Japanese version of the LSNS‐6 has been validated for reliability and accuracy [11]. Social isolation was defined as a score of less than 12 points.

### 2.5 Statistical Analyses

Descriptive statistics were used to summarize demographic and clinical characteristics based on hearing status. Chi‐squared or Fisher's exact tests were applied for categorical variables, while unpaired t‐tests were used for numerical variables. The adjusted risk ratio (aRR) and 95% confidence intervals (CI) for BPSD associated with HI were calculated using modified Poisson regression analysis, adjusted for the propensity score [12]. Modified Poisson regression was chosen over logistic regression as it is more appropriate for estimating risk ratios when dealing with binary outcomes, particularly when the outcome prevalence is not rare, providing more accurate and interpretable estimates than odds ratios. Variables for the propensity score model were selected based on their potential association with both hearing impairment and BPSD, as identified from previous literature and clinical expertise. The propensity score was estimated using logistic regression, with HI as the dependent variable and sex, age, MMSE score, number of psychotropic medications, dementia subtype, conversation frequency and social isolation as independent variables. Balance diagnostics were conducted by comparing the standardized mean differences (SMDs) of covariates between the HI and non‐HI groups prior to propensity score adjustment. Covariates with SMDs ≥ 0.1 were considered imbalanced and in need of adjustment [13]. After propensity score adjustment using inverse probability of treatment weighting (IPTW), weighted SMDs were recalculated to assess covariate balance. An absolute SMD < 0.2 was considered indicative of acceptable balance after weighting [14]. All participants had complete data for the variables included in the modified Poisson regression analysis; therefore, no imputation or exclusion due to missing data was necessary. All analyses were conducted using Stata software version 18.0 (Stata Corp, TX, USA). Statistical significance was set at *p* < 0.05. However, to account for multiple comparisons in the analysis of the association between HI and each BPSD, Bonferroni correction was applied, adjusting the significance level to *p* < 0.002. This threshold was determined by dividing 0.05 by the number of comparisons (27).

## 3 Results

Among the 167 individuals who consented to participate, 144 participants without missing data for the primary outcomes were included in the analysis (Figure 1). The demographic characteristics of the participants, presented overall and stratified by HI status, are shown in Table 1. Among the participants, 64 individuals (44.4%) were classified as having HI, defined by an average hearing threshold of 40 dB or higher. The HI group was significantly older than the without HI group (mean age 85.1 ± 5.5 vs. 80.8 ± 6.1 years, *p* < 0.001). The proportion of participants taking anti‐dementia medications was significantly lower in the HI group compared to the without HI group (50.0% vs. 68.8%, *p* = 0.022). A positive correlation was observed between average hearing level and age (*r* = 0.459, *p* < 0.001).

**TABLE 1:** Characteristics stratified by overall and hearing impairment status.

Characteristics	Missing	Overall		Pure‐tone average				*p*‐value
				< 40 dB (without HI)		40 dB ≧ (HI)		
		*n* = 144		*n* = 80		*n* = 64		
Age, year		82.7	±6.3	80.8	±6.1	85.1	±5.5	< 0.001
Sex, female		96	(66.7)	57	(71.3)	39	(60.9)	0.192
Education years, year	6	11.4	±2.1	11.7	±2.2	11.0	±1.8	0.066
MMSE score		19.3	±6.0	19.8	±5.4	18.7	±6.5	0.283
Dementia type^a^								
Mild cognitive impairment		41	(28.5)	21	(26.3)	20	(31.3)	0.783
Alzheimer's disease		88	(61.1)	50	(62.5)	38	(59.4)	
Dementia other than Alzheimer's disease		15	(10.5)	9	(11.3)	6	(9.4)	
Medical history								
Stroke		13	(9.0)	6	(7.5)	7	(10.9)	0.474
Otorhinolaryngological disorder^b^		3	(2.1)	2	(2.5)	1	(1.6)	1.000
Comorbidities								
Hypertension		85	(59.0)	44	(55.0)	41	(64.1)	0.272
Diabetes mellitus		24	(16.7)	10	(12.5)	14	(21.9)	0.134
Hyperlipidaemia		57	(39.6)	35	(43.8)	22	(34.4)	0.253
Anti‐dementia drug usage^c^		87	(60.4)	55	(68.8)	32	(50.0)	0.022
Psychotropic usage		19	(13.2)	13	(16.3)	6	(9.4)	0.226
Number of psychotropic medications		1.4	±0.7	1.5	±0.8	1.3	±0.5	0.719
SSRI^d^		6	(31.6)	4	(30.8)	2	(33.3)	1.000
Non‐SSRI^e^		8	(42.1)	5	(38.5)	3	(50.0)	1.000
Typical antipsychotic^f^		2	(10.5)	1	(7.7)	1	(16.7)	1.000
Atypical antipsychotic^g^		7	(36.8)	5	(38.5)	2	(33.3)	1.000
Antianxiety drug^h^		2	(10.5)	2	(15.4)	0	(0.0)	1.000
Antiepileptic drug^i^		2	(10.5)	2	(15.4)	0	(0.0)	1.000
Long‐term care insurance status	5							
Not applied		29	(20.1)	21	(27.3)	8	(12.9)	NA
Under application		3	(2.1)	2	(2.6)	1	(1.6)	
Certified for long‐term care		107	(74.3)	54	(70.1)	53	(85.5)	
Living alone		32	(22.2)	18	(22.5)	14	(21.9)	0.929
Daily conversation		130	(90.3)	71	(88.8)	59	(92.2)	0.489
Social isolation		77	(53.5)	40	(50.0)	37	(57.8)	0.350
Social interactions		79	(54.9)	44	(55.0)	35	(54.7)	0.970

*Note:* Values are presented as *n* (%) unless otherwise noted. Chi‐squared tests or Fisher's exact tests were employed for categorical variables, whereas unpaired *t*‐tests were used for numerical variables. Degree of hearing impairment was defined according to the speech‐frequency pure‐tone average of hearing thresholds at 0.5, 1, 2 and 4 kHz in the better‐hearing ear. Based on the Japanese Society of Audiology and World Health Organisation criteria for hearing loss, 40 dB or more was defined as the threshold for hearing impairment.

^a^ Diagnoses at the time of the survey: individuals diagnosed with Alzheimer's disease include those diagnosed with suspected Alzheimer's disease, mild cognitive impairment due to Alzheimer's disease and amnesic mild cognitive impairment.

^b^ Otitis media, otitis fungoides.

^c^ Donepezil hydrochloride, galantamine hydrobromide, rivastigmine, memantine.

^d^ Sertraline hydrochloride, paroxetine hydrochloride hydrate, escitalopram oxalate.

^e^ Mirtazapine, duloxetine hydrochloride.

^f^ Tiapride hydrochloride.

^g^ Aripiprazole, quetiapine fumarate, risperidone.

^h^ Etizolam.

^i^ Sodium valproate, carbamazepine.

The prevalence of each BPSD is presented in Table 2. A total of 139 participants (96.5%) exhibited at least one symptom of BPSD. Following Bonferroni correction, no significant differences were observed between the HI and no‐HI groups in the prevalence of BPSD. However, among the psychological symptoms, anxiety and several behavioural symptoms, including verbally aggressive behaviour, physically aggressive behaviour, leaving home unannounced, inappropriate vocalisation and carelessness with fire, were found to be approximately 2–6 times more prevalent in the HI group. The adjusted risk ratios for each BPSD associated with HI, calculated using modified Poisson regression analysis, are shown in Table 3. The adjusted risk ratios for the presence of each BPSD among the HI group (reference: no‐HI group) were as follows: anxiety had an aRR of 2.08 (95% CI 1.09–3.99), irritability was 1.73 (95% CI: 1.05–2.86), verbally aggressive behaviour was 3.41 (95% CI: 1.24–9.40), physically aggressive behaviour was 5.54 (95% CI: 1.31–23.40), leaving home unannounced was 4.22 (95% CI: 1.25–14.16), hoarding behaviour was 1.83 (95% CI: 1.06–3.16), resistance to care was 2.12 (95% CI: 1.02–4.40) and carelessness with fire was 4.46 (95% CI: 1.12–17.83).

**TABLE 2:** Association between Hearing Impairment and prevalence of BPSD.

	Overall		Without HI		HI		*p*‐value
	*n* = 144		*n* = 80		*n* = 64		
Any symptoms	139	(96.5)	77	(96.3)	62	(96.9)	0.839
Psychological symptoms	75	(52.1)	38	(47.5)	37	(57.8)	0.218
Hyperactive domain							
Visual and auditory hallucinations	21	(14.6)	11	(13.8)	10	(15.6)	0.751
Delusion	39	(27.1)	22	(27.5)	17	(26.6)	0.900
Day‐night reversal	9	(6.3)	4	(5.0)	5	(7.8)	0.511
Hypoactive domain							
Depression	42	(29.2)	24	(30.0)	18	(28.1)	0.806
Anxiety	37	(25.7)	13	(16.3)	24	(37.5)	0.004
Behavioural symptoms	139	(96.5)	77	(96.3)	62	(96.9)	1.000
Hyperactive domain							
Stereotyped behaviour	23	(16.0)	10	(12.5)	13	(20.3)	0.204
Disinhibition	18	(12.5)	9	(11.3)	9	(14.1)	0.612
Changes in eating behaviours	13	(9.0)	7	(8.8)	6	(9.4)	0.897
Irritability	49	(34.0)	23	(28.8)	26	(40.6)	0.135
Verbally aggressive behaviour	17	(11.8)	5	(6.3)	12	(18.8)	0.035
Physically aggressive behaviour	9	(6.3)	2	(2.5)	7	(10.9)	0.078
Wandering and restlessness	28	(19.4)	16	(20.0)	12	(18.8)	0.851
Leaving home unannounced	10	(6.9)	2	(2.5)	8	(12.5)	0.023
Inappropriate sexual behaviour	0	(0.0)	0	(0.0)	0	(0.0)	
Repeated question	128	(88.9)	71	(88.8)	57	(89.1)	0.953
Hording behaviour	41	(28.5)	20	(25.0)	21	(32.8)	0.302
Inappropriate vocalisation	11	(7.6)	4	(5.0)	7	(10.9)	0.217
Resistance to care	29	(20.1)	12	(15.0)	17	(26.6)	0.086
Uncleanliness	19	(13.2)	9	(11.3)	10	(15.6)	0.441
Carelessness of fire	13	(9.0)	3	(3.8)	10	(15.6)	0.018
Hiding and losing things	93	(64.6)	54	(67.5)	39	(60.9)	0.413
Hypoactive domain							
Lack of interest	58	(40.3)	30	(37.5)	28	(43.8)	0.447
Apathy	68	(47.2)	37	(46.3)	31	(48.4)	0.794
Withdrawal	19	(13.2)	12	(15.0)	7	(10.9)	0.474
Somnolence tendency	73	(50.7)	38	(47.5)	35	(54.7)	0.391

*Note:* Values are presented as *n* (%) unless otherwise noted. Chi‐squared tests or Fisher's exact tests were employed for categorical variables. Degree of hearing impairment was defined according to the speech‐frequency pure‐tone average of hearing thresholds at 0.5, 1, 2 and 4 kHz in the better‐hearing ear. Based on the Japanese Society of Audiology and World Health Organization criteria for hearing loss, 40 dB or more was defined as the threshold for hearing impairment. Degree of hearing impairment was defined according to the speech‐frequency pure‐tone average of hearing thresholds at 0.5, 1, 2 and 4 kHz in the better‐hearing ear. Based on the Japanese Society of Audiology and World Health Organization criteria for hearing loss, 40 dB or more was defined as the threshold for hearing impairment. The *p*value was adjusted using the Bonferroni correction and a significance level of *p* < 0.002 was applied.

Abbreviations: BPSD, behavioral and psychological symptoms of dementia; HI, hearing impairment.

**TABLE 3:** Adjusted risk ratios for each BPSD due to HI as calculated using modified Poisson regression analysis.

	Without HI		HI		
	(*n* = 80)		(*n* = 64)		
	aRR	(95% CI)	aRR	(95% CI)	*p*‐value
Any symptoms	(ref.)		1.04	(0.96–1.12)	0.355
Psychological symptoms	(ref.)		1.35	(0.96–1.90)	0.082
Hyperactive domain					
Visual and auditory hallucinations	(ref.)		0.88	(0.36–2.14)	0.777
Delusion	(ref.)		1.03	(0.56–1.89)	0.927
Day‐night reversal	(ref.)		1.46	(0.55–3.89)	0.447
Hypoactive domain					
depression	(ref.)		1.30	(0.76–2.23)	0.335
Anxiety	(ref.)		2.08	(1.09–3.99)	0.027
Behavioural symptoms	(ref.)		1.04	(0.97–1.12)	0.290
Hyperactive domain					
Stereotyped behaviour	(ref.)		1.69	(0.69–4.10)	0.250
Disinhibition	(ref.)		1.70	(0.74–3.94)	0.214
Changes in eating behaviours	(ref.)		1.21	(0.36–4.06)	0.759
Irritability	(ref.)		1.73	(1.05–2.86)	0.032
Verbally aggressive behaviour	(ref.)		3.41	(1.24–9.40)	0.017
Physically aggressive behaviour	(ref.)		5.54	(1.31–23.40)	0.020
Wandering and restlessness	(ref.)		0.97	(0.47–1.97)	0.925
Leaving home unannounced	(ref.)		4.22	(1.25–14.16)	0.020
Repeated question	(ref.)		1.05	(0.92–1.20)	0.455
Hording behaviour	(ref.)		1.83	(1.06–3.16)	0.029
Inappropriate vocalisation	(ref.)		2.15	(0.54–8.59)	0.279
Resistance to care	(ref.)		2.12	(1.02–4.40)	0.044
Uncleanliness	(ref.)		1.71	(0.65–4.47)	0.277
Carelessness of fire	(ref.)		4.46	(1.12–17.83)	0.034
Hiding and losing things	(ref.)		1.02	(0.78–1.33)	0.888
Hypoactive domain					
Lack of interest	(ref.)		1.06	(0.68–1.66)	0.781
Apathy	(ref.)		1.13	(0.76–1.67)	0.543
Withdrawal	(ref.)		0.97	(0.38–2.45)	0.948
Somnolence tendency	(ref.)		1.00	(0.70–1.43)	0.997

*Note:* The adjusted risk ratio for BPSD due to hearing impairment was calculated using a modified Poisson regression analysis, adjusted for the propensity score. The propensity score was estimated using logistic regression, with hearing impairment as the dependent variable and sex, age, MMSE score, number of psychotropic medications, dementia subtype, conversation frequency and social isolation as independent variables. The degree of hearing impairment was defined according to the speech‐frequency pure‐tone average of hearing thresholds at 0.5, 1, 2 and 4 kHz in the better‐hearing ear. Based on the Japanese Society of Audiology and World Health Organisation criteria for hearing loss, 40 dB or more was defined as the threshold for hearing impairment.

Abbreviations: aRR, adjusted risk ratio; BPSD, behavioral and psychological symptoms of dementia; CI, confidence interval; HI, hearing impairment.

## 4 Discussion

This study revealed that individuals with HI had a higher prevalence of psychological symptoms, such as anxiety and behavioral symptoms, including irritability, verbally aggressive behavior, physically aggressive behavior, leaving home unannounced, hoarding behavior, resistance to care and carelessness with fire.

Older adults with hearing loss, defined by an average hearing level of 40 dB or higher, often experience difficulties in communication with others. Furthermore, they demonstrate elevated HHIE‐S scores, which include questions assessing whether individuals feel irritated when conversing with others, indicating that HI significantly impacts both the practical and emotional aspects of interpersonal communication [15]. Additionally, they are more likely to develop mental health issues, such as anxiety associated with tinnitus [16] and depression [17], as well as depressive symptoms [18]. These findings are consistent with the results of this study. Furthermore, communication difficulties related to HI can contribute to psychological distress, including discomfort and frustration, where individuals may feel they cannot understand others or perceive that they are being scolded because others speak loudly. The chronic strain of effortful listening and repeated communication breakdowns associated with HI may also heighten irritability in older adults. This irritability can stem from the cognitive fatigue that results from constantly straining to process auditory information, as well as from the social embarrassment and isolation that often accompany communication difficulties. When individuals with HI repeatedly ask for clarification or misinterpret conversations, they may become increasingly frustrated with both themselves and others, leading to a short temper and reactive behaviors. Additionally, communication misunderstandings may result in resistance to care, as individuals may misinterpret caregivers' instructions or intentions, leading to oppositional behaviors during care activities. This irritability and resistance to care can create a cyclical pattern where communication partners become hesitant to engage, further exacerbating social isolation and emotional distress. These experiences can manifest as emotional expressions through verbal or physical aggressive behaviors and resistance to care provision. These findings suggest that when interacting with individuals with cognitive decline and HI, it is essential to adopt a more attentive communication approach, such as confirming their level of understanding. This may help reduce misunderstandings and alleviate potential frustration and resistance to care, ultimately contributing to better management of BPSD.

The observed association between HI and hoarding behavior may be explained by the tendency of individuals with HI to experience heightened anxiety [16] and loneliness [19]. Those who feel anxious or socially isolated may engage in hoarding as a means of obtaining a sense of security. Additionally, reduced auditory input may hinder the proper processing of information from family members and caregivers, leading individuals to believe that certain items ‘might be useful someday,’ making it difficult for them to discard possessions and resulting in excessive accumulation.

Leaving home unannounced was assessed using the ‘Inclination to leave their homes/institutions’ and is distinct from wandering. A systematic review has reported that hearing loss, regardless of whether it is assessed subjectively or objectively, is associated with social isolation and social withdrawal [19]. However, no studies have specifically examined the relationship between HI and leaving home unannounced. Difficulty hearing environmental sounds may impair an individual's ability to assess situations and, when combined with cognitive decline, could increase the risk of unplanned departures. Moreover, HI could contribute to disorientation, leading to impaired judgment and making individuals with HI more prone to social withdrawal or leaving home unexpectedly.

In this study, the group with HI was older and a weak positive correlation was observed between average hearing level and age. Based on these findings, it is presumed that many individuals in the HI group had age‐related hearing loss (ARHL). ARHL is typically characterized by a gradual decline in hearing sensitivity, particularly in the high‐frequency range [20]. As a result, individuals with ARHL may be unable to hear important environmental cues, such as alarms from gas stoves, potentially increasing carelessness with fire and fire hazards and challenges in daily living. These findings highlight the necessity of strategies to prevent information deprivation and functional impairments caused by HI. Interventions that incorporate individualized approaches tailored to the specific needs of individuals with HI and cognitive decline may be effective in ensuring a safe and comfortable living environment while respecting their autonomy.

The present study was subject to certain limitations. First, hearing levels in the 500 and 1000 Hz frequency bands may have been worse than the actual levels due to the lack of a soundproof environment during pure‐tone audiometry, which could not fully exclude ambient noise. Therefore, defining HI as a PTA ≥ 40 dB might have led to an overestimation. In this study, the proportion of individuals aged 80 years and older with HI was 97% in men and 85% in women when HI was defined as a PTA of 25 dB or more. These proportions were slightly higher than those reported in previous studies, which estimated HI in 84.3% of men and 73.3% of women [21]. The influence of ambient noise in the examination room and participants' cognitive decline cannot be ruled out. However, the hearing test was conducted in accordance with the noise‐level guidelines for hearing aid compatibility testing, which specify a background noise level of 50 dB A‐weighted sound pressure level or lower. Although the test was not performed in a soundproof room, similar methodologies have yielded valid results in previous studies [22, 23]. Hence, the hearing levels measured in this study were reasonable and the testing method demonstrated a certain degree of accuracy. Furthermore, given that this study confirmed a positive correlation between PTA levels and age, the results suggest that the measurements were reasonably reliable despite not being conducted in a soundproof room.

Second, the BPSD assessment used in this study was subjectively conducted by an attendant, such as family members and friends. The subjective nature of this assessment may have introduced respondent bias, potentially affecting the accuracy of the results. Family members who provide daily care may have overestimated BPSD, whereas non‐cohabiting family members or friends who only accompany participants to the clinic may have underestimated BPSD.

Third, despite including age as a covariate in the propensity score model, the significant age difference between groups may not be fully addressed by our adjustment approach, as older participants with HI could independently exhibit a higher prevalence of BPSD. Additionally, the wide confidence intervals observed in some analyses suggest potentially unstable estimates due to the limited sample size. Although no statistically significant associations were found after multiple comparison correction, this should be interpreted cautiously as it may reflect insufficient statistical power rather than the absence of true associations. Furthermore, although social interaction and family composition were balanced between the groups in this study and, therefore, not included as confounding factors, these variables could potentially distort the association between HI and BPSD based on previous research on background factors. This represents a limitation of the current study. Future studies with larger sample sizes are warranted to better control for age‐related confounding factors and to provide more precise estimates.

Finally, this study calculated the aRR for BPSD associated with HI using a modified Poisson regression analysis. However, since multiple hypothesis testing increases the risk of type I errors, the false discovery rate (FDR) correction was applied to control the proportion of false discoveries among significant results. Specifically, the Benjamini‐Hochberg procedure was used to adjust *p* values, ensuring that the expected proportion of incorrectly rejected null hypotheses remained below a specified threshold of 10% (FDR = 0.1). As a result, some findings that were initially significant in the modified Poisson regression analysis were no longer statistically significant. This suggests that some associations observed in the initial analysis may have been due to chance, underscoring the importance of cautious interpretation of the results. However, based on findings from previous studies on the impact of HI, the results obtained in this study appear to be plausible and consistent with existing evidence. These findings highlight the importance of tailored interventions to improve communication strategies and environmental modifications for individuals with HI and cognitive decline, potentially mitigating the impact of BPSD.

In conclusion, present results revealed that HI was associated with a higher prevalence of psychological symptoms, such as anxiety and behavioral symptoms, including irritability, verbally aggressive behavior, physically aggressive behavior, leaving home unannounced, resistance to care, hoarding behavior and carelessness with fire.

## Disclosure

The authors have nothing to report.

## Ethics Statement

This study was conducted in accordance with the ethical standards of the 1975 Declaration of Helsinki and its subsequent amendments or comparable ethical guidelines available at https://www.wma.net/what‐we‐do/medical‐ethics/declaration‐of‐helsinki/. Written informed consent was obtained from all participants and their attendants. Most attendants were family members, with one participant accompanied by a friend. This study was approved by the Ethics Committee of Shiga University of Medical Science (Reference Number: R2023‐009) and the Kyoto Clinical and Translational Research Centre for Neurocognitive Disorders (Reception Number: 23001).

## Conflicts of Interest

The authors declare no conflicts of interest.

## Data Availability

The data that support the findings of this study are available on request from the corresponding author. The data are not publicly available due to privacy or ethical restrictions.
